# Bioactive Properties, Bioavailability Profiles, and Clinical Evidence of the Potential Benefits of Black Pepper (*Piper nigrum*) and Red Pepper (*Capsicum annum*) against Diverse Metabolic Complications

**DOI:** 10.3390/molecules28186569

**Published:** 2023-09-11

**Authors:** Phiwayinkosi V. Dludla, Ilenia Cirilli, Fabio Marcheggiani, Sonia Silvestri, Patrick Orlando, Ndivhuwo Muvhulawa, Marakiya T. Moetlediwa, Bongani B. Nkambule, Sithandiwe E. Mazibuko-Mbeje, Nokulunga Hlengwa, Sidney Hanser, Duduzile Ndwandwe, Jeanine L. Marnewick, Albertus K. Basson, Luca Tiano

**Affiliations:** 1 Cochrane South Africa, South African Medical Research Council, Tygerberg 7505, South Africa; mn.muvhulawa@gmail.com (N.M.); duduzile.ndwandwe@mrc.ac.za (D.N.); 2 Department of Biochemistry and Microbiology, University of Zululand, KwaDlangezwa 3886, South Africa; hlengwan@unizulu.ac.za (N.H.); bassona@unizulu.ac.za (A.K.B.); 3 Department of Life and Environmental Sciences, Polytechnic University of Marche, 60131 Ancona, Italy; ilenia.cirilli@unicam.it (I.C.); f.marcheggiani@univpm.it (F.M.); s.silvestri@univpm.it (S.S.); p.orlando@univpm.it (P.O.); l.tiano@staff.univpm.it (L.T.); 4 Department of Biochemistry, North-West University, Mafikeng Campus, Mmabatho 2735, South Africa; mtdmoetlediwa@gmail.com (M.T.M.); sithandiwe.mazibukombeje@nwu.ac.za (S.E.M.-M.); 5 School of Laboratory Medicine and Medical Sciences, University of KwaZulu-Natal, Durban 4000, South Africa; nkambuleb@ukzn.ac.za; 6 Department of Physiology and Environmental Health, University of Limpopo, Sovenga 0727, South Africa; sidney.hanser@ul.ac.za; 7 Applied Microbial and Health Biotechnology Institute, Cape Peninsula University of Technology, Bellville 7535, South Africa; marnewickj@cput.ac.za

**Keywords:** metabolic disease, oxidative stress, inflammation, pepper, piperine, capsaicin, capsinoid

## Abstract

The consumption of food-derived products, including the regular intake of pepper, is increasingly evaluated for its potential benefits in protecting against diverse metabolic complications. The current study made use of prominent electronic databases including PubMed, Google Scholar, and Scopus to retrieve clinical evidence linking the intake of black and red pepper with the amelioration of metabolic complications. The findings summarize evidence supporting the beneficial effects of black pepper (*Piper nigrum* L.), including its active ingredient, piperine, in improving blood lipid profiles, including reducing circulating levels of total cholesterol, low-density lipoprotein cholesterol, and triglycerides in overweight and obese individuals. The intake of piperine was also linked with enhanced antioxidant and anti-inflammatory properties by increasing serum levels of superoxide dismutase while reducing those of malonaldehyde and C-reactive protein in individuals with metabolic syndrome. Evidence summarized in the current review also indicates that red pepper (*Capsicum annum*), together with its active ingredient, capsaicin, could promote energy expenditure, including limiting energy intake, which is likely to contribute to reduced fat mass in overweight and obese individuals. Emerging clinical evidence also indicates that pepper may be beneficial in alleviating complications linked with other chronic conditions, including osteoarthritis, oropharyngeal dysphagia, digestion, hemodialysis, and neuromuscular fatigue. Notably, the beneficial effects of pepper or its active ingredients appear to be more pronounced when used in combination with other bioactive compounds. The current review also covers essential information on the metabolism and bioavailability profiles of both pepper species and their main active ingredients, which are all necessary to understand their potential beneficial effects against metabolic diseases.

## 1. Introduction

**Figure 1:**
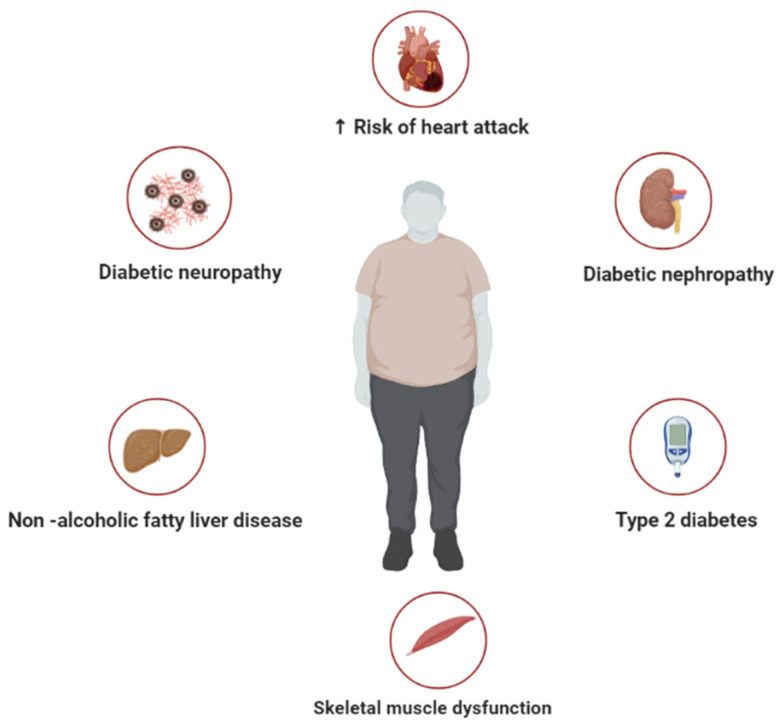
A general overview of metabolic syndrome, representing some of the diverse pathological conditions associated with this abnormal metabolic state, including diabetic neuropathy, type 2 diabetes, non-alcoholic fatty liver disease, skeletal muscle dysfunction, and increased risk of heart failure.

Metabolic syndrome describes a cluster of metabolic complications, including insulin resistance, hypertension, and hyperlipidemia, that increase the risk for the development of cardiovascular diseases [1,2]. Cardiovascular diseases remain the leading cause of death worldwide [3], especially in people with metabolic disorders [4]. Recent numbers indicate that a growing number of individuals present with a cluster of metabolic disorders such as hyperglycemia and dyslipidemia that are also linked with the development and progression of type 2 diabetes [5]. This emphasizes an urgent need for multisectoral interventions to decrease the global burden of metabolic syndrome and associated complications, especially those involving overweight and obesity (Figure 1). Indeed, the consumption of a high-calorie diet, in combination with reduced physical activity or a sedentary lifestyle, is known to be the major cause of obesity that accelerates the development of metabolic syndrome [6]. An obese state is accompanied by excessive adiposity and enhanced ectopic accumulation, which is associated with increased levels of oxidative stress and inflammation [7]. Both oxidative stress and inflammation are considered prominent pathological mechanisms that alter biochemical processes and cause cellular damage within many metabolic diseases [8,9].

There has been an increasing interest in evaluating the therapeutic potential of dietary sources, including foods rich in antioxidants, for their ameliorative effects against oxidative stress and inflammation in diverse metabolic conditions. In fact, our group and others have progressively reported on the potential benefits of plant- and/or food-derived bioactive compounds for their capacity to improve metabolic status by blocking the toxic effects of oxidative stress and inflammation [10,11,12,13,14]. A growing body of literature has also progressively reported on the potential benefits of pepper against diverse metabolic complications [15,16,17,18]. *Piper*, the genus of pepper plants or pepper vines, is contemplated to be part of the most ancient pan-tropical flowering plant groups [19]. With an estimated 1000 species of herbs, encompassing small trees, shrubs, and hanging vines, the genus *Piper* is considered to have a rich ethnobotanical and ethnopharmaceutical history [20]. The reviewed literature already indicates the potential therapeutic effects of pepper; however, it predominantly focuses on preclinical findings [15,16,17,18]. In particular, reviewed information shows that black and red pepper, including their respective main bioactive compounds piperine and capsaicin, display a variety of biological effects, including antimicrobial, anti-inflammatory, gastro-protective, antidepressant, and antioxidant properties, in preclinical models [16,17]. Azlan and colleagues also recently reviewed evidence of the antioxidant and anti-obesity effects of different chili peppers [18]. However, a gap remains in the evidence on the clinical benefits of pepper against metabolic diseases. Importantly, there are no reviews that have compared the therapeutic effects of both black pepper (*Piper nigrum*) and red pepper (*Capsicum annum*) against metabolic diseases. This highlights the importance of the current review, which critically discusses clinical evidence of the potential benefits of both black and red peppers against diverse metabolic complications. The current review also covers essential information on the biological properties, metabolism, and bioavailability profiles, as well as the toxic effects, of pepper types and their main active ingredients, which are all necessary to underscore its potential pharmacological relevance.

## 2. General Overview of Black Pepper (*Piper nigrum*), including Its Metabolism and Bioavailability Profile

**Figure 2:**
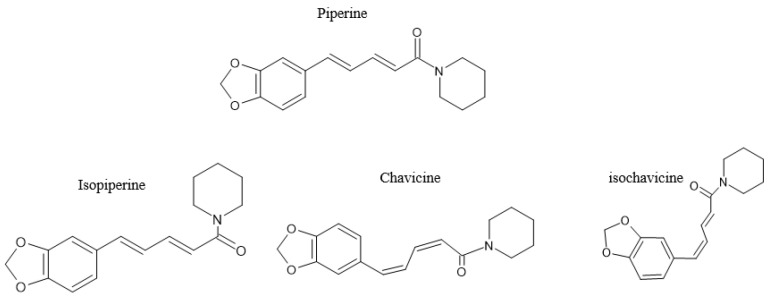
The chemical structure of piperine, including its isomers isopiperine, chavivine, and isochavicine; adapted from published literature [34].

The genus *Piperaceae*, of the pepper family, contains flowering plants including small trees, shrubs, or herbs. This class consists of about 3600 species and five genera, including *Piper*, *Peperomia*, *Zippelia*, *Manekia*, and *Verhuellia*. Most of the species are found in the *Piper* genera, with about 2171 species, and *Peperomia*, with over 1000 species [21]. The most popular species of the *Piperaceae* family is *Piper nigrum*, which produces peppercorns that are generally used as spices, including black pepper, which is considered the king of spices [22]. Another well-known species of the *Piperaceae* is *Piper longum*, which yields black, white, and green peppercorns [23]. It is believed that the Piper genus is endogenous to India [24], being broadly cultivated within the Karala region [25]. Black pepper contains a major bioactive pungent alkaloid commonly known as piperine, which is found in the fruits of *Piper longum* and *Piper nigrum* [23]. Piperine content within black pepper is estimated to range from 2–10% [26,27,28,29,30,31]. Piperine was first extracted around the 1800s, while its chemical composition was elucidated much later, around 1882–1894 [32]. Since then, research has extensively studied black and its constituent piperine, with the description of different isomers of the bioactive compound currently acknowledged, including the trans–trans isomer (piperine), cis–trans isomer (isopiperine), cis–cis isomer (chavicine), and trans–cis isomer (isochevicine) (Figure 2). Other alkaloids that have been identified in black pepper include piperanine, piperettine, piperolein, piperylin, and pipericine [22]. Reviewed information already indicates that other bioactive compounds can be found in the African *Piper* species [33]. Like other natural compounds that are widely ingested orally, piperine gets broken down within the body into small components or metabolites.

Evidence from animal studies shows that oral administration of 170 mg of piperine yields approximately 3–4% of the original ingested bioactive compounds, which is detected mainly in feces after 4 or 5 days in rats, while about 96–97% is projected to be absorbed [35,36]. Ingested piperine is normally absorbed within the small intestines of rats [35]. When 170 mg of the bioactive compound is given to rats, about 38.8 µmol of piperine is detected in the serum, while some of the trace elements of the compound are found in the liver and kidneys [36]. Although some studies could not detect the presence of piperine in urine or serum [35,36,37,38], others have reported piperine metabolites including piperonylic acid, piperonyl alcohol, piperonal, and their conjugates in the urine of rats [39]. Piperine, when conjugated with iron, can inhibit the activity of CYP450 3A4 [40], an essential enzyme involved in drug metabolism and detoxication processes within the liver [41]. Additional evidence indicates that twelve metabolites of piperine can be detected in rat plasma, bile, feces, and urine [42]. Apparently, piperine can undergo a series of chemical modifications through the enzymes responsible for the liver first-pass metabolic effect [43]. Studies support effective absorption of piperine in rats, especially after an initial oral dose of 20 mg is given [44]. Others have also affirmed effective absorption of piperine through enhanced levels in the brain and plasma of rats after the ingestion of a dose of 35 mg [45]. The metabolism of 50 mg of piperine in humans translates to about 0.71–0.83 mg of the bioactive compound being detected within the plasma [46]. Importantly, piperine metabolites, including 5-(3-4-dihydroxphenyl) valeric acid piperidide and its derivative 5-(3-4-dihydroxphenyl)valeric acid-4-hydroxypiperidide, have been detected in the urine of humans [47].

Like with other natural compounds, the delivery of piperine is supposedly compromised by its low water solubility, which could lead to poor clinical applications [48]. However, research has evaluated the potential use of delivery systems like nanoparticles, nanoliposomes, and micelles among advances to improve the bioavailability of natural bioactive and food compounds [49]. For example, the encapsulation of piperine in a nanoparticle of sodium chitosan triphosphate could improve its absorption, leading to enhanced biological activity [50]. A combination of piperine and curcumin shows improved efficacy compared to that of each bioactive compound alone [51]. Others have indicated that the use of mixed micelles of D-alpha tocopherol polyethylene glycol succinate and soluplus could improve the efficacy of encapsulated piperine over that of free piperine [52]. Cubic-nanoparticle-encapsulated piperine and protopanaxadiol also show improved bioavailability [53]. Such studies indicate that piperine is released at a much faster rate within in vivo systems, while also promoting increased absorption. Another interesting formulation is a nanoliposome with piperine and gentamicin, as investigated in bacterial growth [54]. It has been found that this liposomal combination was effective in inducing death and bacterial inhibition. Lastly, other researchers have used solid lipid nanoparticles encapsulating piperine to test the ameliorative effect of piperine against the complications of Alzheimer’s disease [55]. Interestingly, it was found that this nano-formulation could reduce the levels of superoxide dismutase (SOD) as well as oxidative stress at a dose of 2 mg/kg [55]. This further indicates that the bioavailability profile or bioactivity of piperine can be enhanced through recent developments in drug discovery, especially when used in combination with other bioactive compounds or food products [56,57].

## 3. Red Pepper (*Capsicum annum*), including Its Metabolism and Bioavailability Profile

Red pepper belongs to the Solanaceae family of the genus *Capsicum*, consisting of five domesticated species such as *C. annuum* L., *C. chinense* Jacq., *C. frutescens* L., *C. baccatum* L., and *C. pubescens* Ruiz et Pav [58,59]. It appears the capsicum species were first discovered in Bolivia, with their cultivation expanding to Mexico prior to the Columbian times (7000 B.C.) [60]. The capsicum genus is known by different names including hot pepper, chili pepper, bell pepper, sweet pepper, and sometimes just pepper across the world. Red pepper consists of different secondary metabolites collectively called capsaicinoids [61]. Capsaicinoids include capsaicin (8-methyl-*N*-vanillyl-6-nonenamide) and homologs of capsaicin with acid amides of vanillyl amine, as well as 8–to–18 carbon fatty acids. Other capsaicinoids that exist in pungent red pepper apart from capsaicin include the 6,7-dihydro analog of capsaicin, called dihydrocapsaicin, and nordihydrocapsaicin, which contains the mono-nor homolog of the acyl residue of dihydrocapsaicin [62].

**Figure 3:**
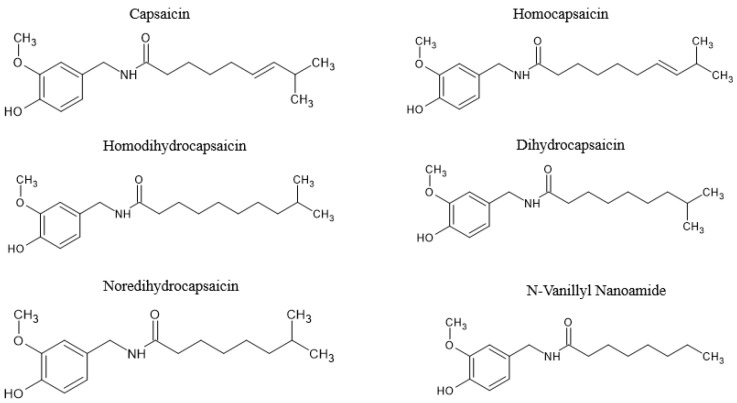
The chemical structure of capsaicinoids, including capsaicin, homocapsaicin, homodihydrocapsaicin and *N*-vanillyl nanoamide. Information adapted from previous literature [63,74].

Figure 3 shows some homo-capsaicinoids, including homocapsaicin, homodihydrocapsaicin and *N*-vanillyl nanoamide [63]. However, capsaicin, a major bioactive capsaicinoid that occurs as a colorless and odorless hydrophobic compound and is crystalline to waxy [64], is mainly responsible for the burning sensation of the fruit when orally ingested [65]. It has been reported that capsaicin and dihydrocapsaicin make up about 70–90% of the capsaicinoids within the *Capsicum* genus [66,67]. Studies have also discovered other capsaicinoid-like residues that are structurally related to capsaicin but of the non-pungent cultivar of *Capsicum annuum* L. [68]. The other structures of these capsaicinoid-like residues have been identified and denoted as capsiate, dihydrocapsiate, and nordihydrocapsiate, with the chemical nomenclature 4-hydroxy-3-methoxybenzyl [E]-8-methyl-6-nonenoate, 4-hydroxy-3-methoxybenzyl 8-methyloctanoate, and 4-hydroxy-3-methoxybenzyl, respectively [68,69]. Structurally, these capsinoids differ from capsaicin by their two moieties that are linked by an ester bond rather than an amine bond [70], and they are called capsinoids [69]. Studies on the pungent components of capsicum with different names such as capsicol, capsaicin, and capsaicin began as early as the 1800s [71]. This is almost the same time when capsaicin was extracted from *Capsicum* [72], and its chemical composition was then characterized by the 1900s [73].

Just like piperine, capsaicin also undergoes liver first-pass metabolism upon oral administration [75]. Evidence indicates that capsaicin can undergo hepatic metabolism, leading to the generation of metabolites like hydroxycapsaicin and dihydrocapsaicin [76]. Apparently, five metabolites of capsaicin have been detected in the human liver, with hydroxycapsaicin, hydroxycapsaicin, and dehydrocapsaicin being the most abundant [77]. Accordingly, the most abundant metabolites in the liver fractions of rats included vanillylamine, hydroxycapsaicin, and dehydrocapsaicin [77]. This finding indicates that capsaicin is metabolized at a higher rate within the liver of rodents. Others have indicated the distribution of capsaicin in different organs of rats, showing that this bioactive compound is abundantly found in the spinal cord and the brain when compared to levels in the liver after intravenous administration [75]. It has also been indicated that reduced levels of capsaicin within the liver may be due to the detoxification process, which produces tolerable conjugates with glucuronic acid or sulfuric acid [75]. This hypothesis has been confirmed by others showing that hydrocapsaicin and other metabolites are found in the urine and feces of rats [71]. It appears that hydrocapsaicin is mainly hydrolyzed by the liver to yield fatty acids and vanillylamine, which are later reduced to vanillin and lastly vanillic acid and/or vanillyl alcohol. Dicapsaicin is another identified metabolite of capsaicin within the liver of rats [78]. Apparently, the P450 enzymes can metabolize capsaicin to produce free-radical intermediates. Other studies have also reported the involvement of a monooxidase system in livers treated with capsaicinoids. For instance, it was previously reported that capsaicinoids were converted to *N*-(4,5,dihydroxy-3-methoxybenzy1) acylamides in rat livers through a mixed-function monooxidase system promoted by hexobarbital injection [71]. In vitro, it was shown that *N*-(4,5,dihydroxy-3-methoxybenzy1) acylamide was the only metabolite detected in the incubation medium containing rat liver homogenate and capsaicin [71].

In terms of bioavailability, intestinal absorption of capsaicin in rats and hamsters in vitro has been reported [79]. From these results, it was evident that hamsters had better capsaicin intestinal absorption than rats [71]. Capsaicinoids are absorbed better in the stomach than in the small intestine in vivo, while in vitro evidence through intestinal sacs also supports enhanced intestinal absorption of capsaicin [79]. Capsaicin can also be better absorbed by the jejunum and ileum than by the stomach in rats [80]. The absorption of capsaicin in the lungs appears to be 20–40-fold slower than that in the liver microsomes of both rats and humans [76]. However, the capsaicin metabolites observed in human lung microsomes were similar to those in liver microsomes. Notably, the metabolic profile of red pepper and capsaicin has been poorly investigated in clinical subjects.

With a rapid advancement in science, nanotechnology has been employed as a potential delivery system to enhance the bioavailability of capsaicin. In this context, polymeric nanocapsules have been used to improve the efficacy of capsaicin [81]. Such data has verified that capsaicin is insoluble in water; thus, the introduction of a high-water emulsion reduces its loading efficacy. Solid-lipidic nanoparticles and nanostructured lipid carriers have also been investigated as transdermal transporters of capsaicin. It has been reported that this delivery emulsion exhibits enhanced transdermal permeability and retention in mice skin [82]. Like most of the formulations, the nano-vascular ethosomal formulation was found to improve permeability in ex vivo human skin and improved the anti-arthritic form of capsaicin [83,84]. Also, there has been interest in exploring the use of nanofibers as a capsaicinoid transdermal delivery strategy. A nanofiber was loaded 0.5–2% of capsaicin extract, and it was demonstrated that the release of this bioactive compound and its permeability in snakeskin were high [85]. Lastly, researchers attempted to encapsulate capsaicin in nanoparticles and incorporate chitosan hydrogel to improve its permeability through the skin [86]. This study compiled as many of the formulation strategies for capsaicin delivery systems as possible. Ongoing research continues to cover the different formulations that can be used to enhance the absorption of capsaicin [63].

## 4. Traditional Uses and Proposed Pharmacological Properties of Pepper and Its Bioactive Compounds

Black pepper and piperine have been acknowledged to have beneficial effects on human health. Starting with preclinical evidence, it has been shown that the administration of piperine at a dose of 50 mg/kg could improve the digestive system while reducing oxidative stress and inflammation in mice [87]. In diverse experimental models of chronic diseases, it was shown that piperine can reduce complications of arthritis [88], hepatic steatosis [89,90], and type 2 diabetes or obesity [91]. It was disclosed that piperine can also reduce depression in mice when given at doses of 2.5, 5, or 10 mg/kg for 14 days [92,93]. Reviewed information has also covered the beneficial effects of black pepper and piperine in various experimental models of disease [94,95]. The molecular mechanisms and signaling pathways that are associated with the ameliorative effects of piperine against the toxic effects of oxidative stress have been discussed, and these include the activation of nuclear factor erythroid 2-related factor 2, peroxisome proliferator-activated receptor-gamma, cyclooxygenase-2, and nitric oxide synthases-2, which is essential to promote intracellular antioxidant responses [96,97]. This bioactive compound can also block inflammation and improve cellular function by effectively modulating or inhibiting multiple signaling pathways, such as those of protein-kinase-activated NLR family pyrin domain containing-3 inflammasome, nuclear factor-κB, Jun *N*-terminal kinase/p38 mitogen-activated protein kinase, and pro-inflammatory molecules [96,98].

On the other hand, red pepper has been used traditionally to relieve toothache. Other traditional uses of red pepper include its application as a home remedy to heal lung conditions like bronchitis, lower glucose levels in diabetes, stabilize blood pressure, and relieve burning feet [99,100]. Scientific evidence indicates that red pepper can improve blood circulation and gastric abnormalities [101] while ameliorating neuralgia and rheumatism [102,103]. Capsaicin and its derivatives are effective against abdominal pain, bloating [104], and pain [105,106,107], as well as alleviating other complications that underlie diabetes and overweight [108]. In addition, it has been shown that capsaicin alone has anti-inflammatory [109] and antioxidant [110,111,112] properties. Other reviews have also given a general perspective into the diverse biological activities of capsaicin, especially in relation to the alleviation of metabolic-disease-related complications [16,63]. It appears that the secondary metabolites of red pepper are equally important in improving human health [113]. In terms of molecular insights, Caterina and colleagues [114] were fundamental in discovering the role of capsaicin as an analgesic agent. Their findings affirmed that capsaicin receptor is a non-selective cation channel that is structurally linked to members of the TRP family of ion channels. The latter encodes integral membrane proteins that function as ion channels and are broadly expressed in diverse tissues and cell types, where they are involved in different physiological processes, including sensation of different stimuli or ion homeostasis [115]. As a result, accumulative research has explored the potential role of capsaicin in stimulating painful sensations, particularly its chemical modulation of sensory neurons through the vanilloid receptor subtype 1 [116,117,118]. Other studies show that mice lacking TRPV1 exhibit no vanilloid-induced pain behavior, which is related to a reduced capacity to feel pain [117].

## 5. Potential Toxic Effects of Pepper

An increasing body of evidence shows that pepper has toxicological effects when used at very high doses. In fact, although considered beneficial to human health, even black pepper is a culprit for such toxic effects. For instance, the administration of its active ingredient, piperine, at doses as high as 60 mg/kg could be lethal in female rats, while a dose of 35.5 mg/kg could be toxic in weaning male rats, although this could also be dependent on prolonged exposure to the compound [119]. It appears that doses of piperine ranging from 35.7 mg/kg–140 mg/kg administered orally could cause liver damage, with 140 mg/kg also affecting kidneys and lungs in mice [120]. Also, high doses of piperine could affect sperm quality in rats [121]. Several authors have also reported that piperine can negatively influence maternal reproduction in association with embryonic toxicity in various preclinical models [122,123,124]. However, more work is required to determine the role of dose dependence, as well as intervention period, in driving the toxic effects of piperine or black pepper. Although piperine has also been studied in humans, it is noteworthy that there is lack of information on its toxicity profile. On the other hand, it has been shown that red pepper constituents (capsaicinoids) could induce skin irritation and inflammation in the mucus and eyes [125]. It has been previously reported that high quantity of capsaicinoids have severe effects on the gastrointestinal tract [126,127]. Since the identification of capsaicinoid toxicity, studies have elucidated the lethal dose of capsaicin in mice, which could be about 122–294 mg/kg, whereas a lethal intravenously injected dose was predicted to be 0.36–0.87 mg/kg [128]. In rats, capsaicin was found to damage the liver mitochondria [129,130]. Apart from being fatal, capsaicin was also reported to suppress stimulus response and induce neurotoxicity, mainly when administered in neonates [130,131,132]. In humans, capsaicin at 0.006% is routinely used to induce a burning stimulus [133]. The intolerable effects of capsaicin could include coughing, diarrhea, and vomiting [134]. Also, capsaicin plasters containing 345.8 mg and 34.58 mg tinctures were shown to induce pain and nausea [135]. There is a large body of knowledge on capsaicin toxicity that has been reported elsewhere [71,74,136].

## 6. Available Clinical Evidence of the Potential Benefits of Pepper

### 6.1. Characteristic Features of Clinical Studies

To identify relevant clinical studies, a systematic search was conducted using major electronic databases including PubMed, Scopus, and Google Scholar. The search strategy was compiled using the following keywords or Medical Subject Headings (MeSH): “pepper” and “metabolic diseases”, including most relevant synonyms as well as keywords related to the search topic. The literature search was performed from inception until June 2023, while a manual search was performed to identify additional relevant studies. The final search results yielded 14 relevant studies reporting on black pepper or its main active ingredient, piperine, and its potential therapeutic effects against diverse metabolic complications (Table 1), whereas 16 records were identified for clinical studies on red pepper, including its active ingredients, capsinoids, against diverse metabolic complications. Besides those from Argentina, Australia, Brazil, China, India, and Japan, which were outliers, most included studies were from Iran, Europe, and the United States, predominantly focusing on adults over the age of 18 years (Table 1). Summarized literature mainly included overweight and obese subjects and those with metabolic syndrome (Table 1 and Table 2). However, evidence involving healthy subjects was also included, provided it was reporting on the therapeutic effects of pepper or its active ingredients on metabolic parameters in individuals with chronic or metabolic conditions.

### 6.2. Evidence of the Effects of Pepper on Overweight and Obese Individuals

**Figure 4:**
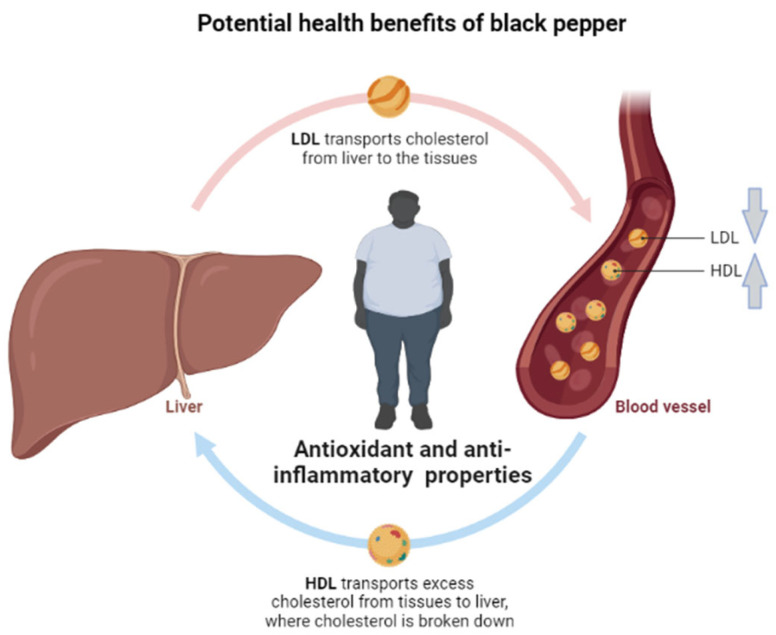
A general overview of the potential beneficial effects of black pepper against obesity and its associated complications, with evidence indicating that black pepper, including its active ingredient piperine, shows enhanced potential to improve blood lipid profiles, including reducing circulating levels of low-density lipoprotein cholesterol (LDL) while increasing those of high-density lipoprotein (HDL). The strong antioxidant properties of black pepper are attributed to its potential beneficial effects on overweight and obese individuals.

Overweight and obesity remain the major contributors to the development of diverse metabolic complications [7,165]. Overnutrition consistent with reduced physical activity is considered the underlying factor driving the development and progression of obesity [166]. In fact, there is an increasing need to investigate the therapeutic effects of pepper against obesity and its associated complications in human subjects. Evidence summarized in this review indicates that several clinical studies have been completed to test the beneficial effects of pepper, including its active ingredients, piperine and capsaicin, on obesity and its related metabolic complications (Table 1). Starting with evidence on black pepper, 3 months of administration of a formulation containing its main ingredient, pure piperine (at 15 mg), together with *Camellia sinensis* soy distearoylphosphatidylcholine was shown to reduce body weight and fat mass in obese subjects [143]. Captivatingly, reviewed evidence already supports the notion that epigallocatechin, which is one of the major active ingredients of *Camellia sinensis*, could potentially neutralize oxidative stress and inflammation to amend complications of metabolic syndrome [165]. The 8-week administration of two capsules containing a combination of piper nigrum dry extract (3 mg), capsicum annum (7.5 mg), and decaffeinated dried *Camellia sinensis* extract (150 mg) could ameliorate obesity-related complications, including insulin resistance, leptin/adiponectin ratio, and low-density lipoprotein (LDL) cholesterol levels, while blocking inflammation in overweight individuals [91]. Similarly, a four-hour administration of a capsule containing a combination of spices (at 2 or 14.5 g) consisting of black pepper, cinnamon, cloves, garlic, ginger, oregano, paprika, rosemary, and turmeric could reduce the levels of triglycerides while alleviating high-fat-meal-induced postprandial interleukin (IL)-1β secretion in overweight and obese subjects [141,147]. Interestingly, an additional study also showed that the beneficial effects of pepper-containing formulations were associated with improved lipid profiles, as seen with reductions in total cholesterol, low-density lipopolysaccharide, and triglyceride levels when individuals with hypercholesterolemia were given capsules comprising different active ingredients such as bioflavonoids, vitamins, omega-3 fatty acids, and black pepper for 30 days [139]. Summarized evidence on the potential therapeutic effects of black pepper against obesity and its associated complications appeared to be more pronounced when used in combination with other active ingredients, with enhanced effects on improving lipid profiles through the reduction of total cholesterol and triglyceride levels, while also lowering pro-inflammatory markers in overweight and obese subjects (Table 1; Figure 4).

Summarized evidence also reported the therapeutic potential of red pepper against obesity and its related complications (Table 2). Here, it was shown that the 7-day administration of a tablet containing an active ingredient of red pepper, capsaicin (at 0.2 mg), together with green tea extract (at 250 mg), tyrosine (at 203 mg), and anhydrous caffeine (at 25.4 mg) could promote a thermogenic effect and enhance energy expenditure in overweight and obese individuals [152]. This is of great importance since it has been estimated that both common therapies like metformin and prominent bioactive compounds can improve metabolic function by promoting thermogenesis and increasing energy expenditure [167,168]. Interestingly, a 4-week administration of capsinoids (at 3 or 10 mg/kg) could also promote fat oxidation, which was positively correlated with enhanced energy expenditure in overweight subjects [154]. Other studies supported the beneficial effects of capsinoids against obesity-related complications, indicating that consuming these bioactive compounds (at doses between 4–6 mg) for 1 to 3 months could promote fat oxidation while reducing body weight, body mass index, and appetite in overweight subjects [70,162]. Moreover, overweight individuals receiving a 4-week chili blend (at 30 g/d; 55%) intervention displayed reduced postprandial hyperinsulinemia [153]. However, administration of a combination of red pepper spices (at 1 g daily for 4 weeks) did not have a significant effect in improving markers of oxidative stress and inflammation in overweight and obese subjects [157]. Individuals subjected to high-fat and high-carbohydrate meals rich in red pepper (at 10 g) exhibited reduced appetite and subsequently reduced protein and fat intakes, while also limiting energy intake [150]. This may indicate that the therapeutic properties of red pepper and its active ingredients, capsinoids, mainly include promoting energy expenditure, including limiting energy intake in overweight and obese individuals.

**Figure 5:**
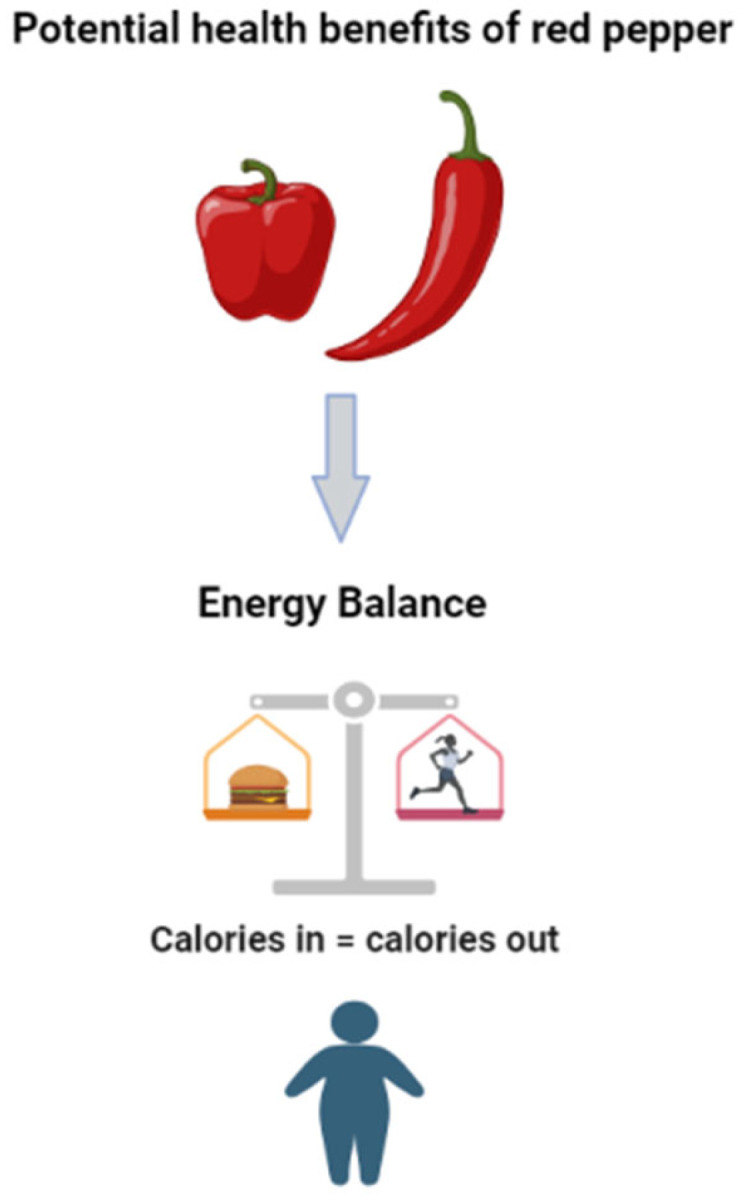
The potential beneficial effects of red pepper, including its active ingredient capsaicin, could promote energy expenditure and limit energy intake, which is likely to contribute to reduced fat mass in overweight and obese individuals.

This was also verified independently in healthy individuals, where the short-term (at least 30 min) administration of capsinoids (at 10 mg) could enhance adrenergic activity and energy expenditure, leading to a shift in substrate utilization toward lipids at rest, but with little effect during exercise or recovery [156]. Also, individuals receiving capsaicin at a dose of 2.56 mg (1.03 g of red chili pepper) with every meal for 36 h displayed a negative energy balance that was concomitant with a reduction in energy expenditure [158]. Similar results were seen for individuals taking red chili pepper containing capsaicin at 2484 µ/g, nordihydrocapsaicin at 278 µ/g, and dihydrocapsaicin at 1440 µ/g (2.56 mg) in terms of enhancing satiety and fullness while preventing a negative energy balance and desire to eat [159]. Interestingly, the positive effects of black pepper on energy expenditure were very minimal [137,138,144]. In fact, very limited information [160] showed that pepper or its active ingredients could affect or improve the metabolic status of individuals with diverse metabolic complications. The potential beneficial effects of red pepper are summarized in Figure 5.

### 6.3. Evidence of the Effects of Pepper on Individuals with Metabolic Syndrome

Overweight and obesity are currently acknowledged to be the leading factors that favor the development of metabolic syndrome. This is a chronic medical condition that describes a cluster of metabolic abnormalities that drive the development of type 2 diabetes and cardiovascular complications. Well beyond assessing the therapeutic effects of pepper against overweight or obesity, it is also important to uncover whether administration of this dietary compound can alleviate the pathological features associated with metabolic syndrome. In fact, some of the evidence included within this review did report the potential benefits of pepper or its active ingredients in complications of metabolic syndrome. (Table 1 and Table 2). For instance, an 8-week co-administration of piperine (at 10 mg) with curcuminoids (at 1 g) could improve oxidative and inflammatory status by enhancing serum levels of SOD while reducing those of malonaldehyde (MDA) and C-reactive protein in individuals with metabolic syndrome [142]. Of course, lipid peroxidation through enhanced levels of MDA is one of the predominant features driving the complications of metabolic disease [169]. It was also shown that a 3-month administration of a formulation containing piperine (at 5 mg) together with resveratrol (at 50 mg) and alpha tocopherol (at 25 mg) could alleviate inflammation by reducing levels of ferritin and C-reactive protein in individuals with metabolic syndrome [148]. The short-term (2 h) administration of capsaicin (at 5 mg) could also reduce plasma glucose levels and maintain insulin levels in individuals subjected to oral glucose tolerance tests [155]. A 4-week administration of a similar dose of capsaicin was also shown to improve plasma postprandial hyperglycemia and hyperinsulinemia in women with gestational diabetes [161]. Notably, limited evidence has directly evaluated the beneficial effects of pepper against the complications linked with metabolic syndrome, while active ingredients such as piperine and capsaicin show the therapeutic potential to improve oxidative stress and inflammation within such pathological conditions.

### 6.4. Evidence of the Effects of Pepper on Individuals with Metabolic Syndrome

Many other chronic conditions contribute to the development and progression of noncommunicable diseases. The current study also evaluated the therapeutic effects of pepper against diverse chronic conditions, including those involving osteoarthritis, oropharyngeal dysphagia, digestion, hemodialysis, and neuromuscular fatigue (Table 1 and Table 2). For instance, a 4-week administration of a formulation containing piperine (at 3.75 mg) together with curcumin (at 300 mg) and gingerols (at 7.5 mg) could potentially protect against chronic knee osteoporosis by reducing levels of prostaglandin E2 [146]. The administration of piperine (at 1 mM or 150 μM) could improve swallowing in individuals with oropharyngeal dysphagia [140]. Those receiving a 2-week intervention with a formulation containing *Trachyspermum ammi* (L.) Sprague seed, *Zingiber officinale* Roscoe. rhizome and *Piper nigrum* L. berry (at 500 mg) presented with improved bloating status, including eructation, defecation, and borborygmus, compared to that seen in individuals treated with dimethicone [145]. Patients undergoing hemodialysis could benefit from receiving a combination of piperine (at 2 mg) and turmeric (at 3 g) for 12 weeks through an effective reduction in biomarkers of oxidative stress and inflammation, including malonaldehyde and ferritin [164]. On the other hand, a reduction in false alarm errors and mental fatigue at different time periods was also reported in young adults with low energy after receiving black pepper capsules twice a day at 0.504 g for 2 days [149]. Individuals receiving two capsules of capsaicin at 390 mg, with 72 h between sessions, displayed improvements in neuromuscular fatigue through alterations in afferent signaling or neuromuscular relaxation kinetics [163]. The blockage of systematic inflammation, leading to amendments in norepinephrine, heart rate, and systolic blood pressure during the experimental task, is the likely mechanism associated with improvements in stressful conditions in individuals receiving capsaicin at 510 mg for 10 days [151].

## 7. Summary and Future Perspectives

For centuries now, spices have been an important part of the human diet. This explains the significant interest directed at understanding the therapeutic benefits of species against many diseases, including the use of pepper and its active ingredients [170]. Clinical evidence covered within the current review supports the beneficial effects of pepper against obesity and its associated complications (Table 1 and Table 2). In fact, summarized literature supports the beneficial effects of black pepper (*Piper Nigrum* L.), including its active ingredient piperine, on lipid profiles, including reducing circulating levels of total cholesterol, low-density lipoprotein cholesterol, and triglycerides in overweight and obese individuals (Table 1). Moreover, the potential therapeutic effects of black pepper and piperine also supposedly include enhanced reduction effects on markers of oxidative stress and inflammation. Interestingly, the literature that has already been reviewed indicates that beyond the strong antioxidant effects, black pepper, with its active ingredient, piperine, contains a rich phytochemistry that includes volatile oil, oleoresins, and alkaloids, giving it the biological properties to protect against the toxic effects of oxidative stress and inflammation [15].

Evidence summarized in the current review also indicates that red pepper, together with its active ingredients, capsinoids, displays enhanced benefits in promoting energy expenditure, including limiting energy intake, which is likely to reduce fat mass in overweight and obese individuals. Although red pepper can potentially improve the oxidative and inflammatory status of individuals with metabolic syndrome, very limited information currently affirms the beneficial effects of both black and red pepper on these individuals. However, preclinical evidence supports the beneficial effects of dietary capsaicin in improving glucose homeostasis and lipid metabolism through the modulation of bile acid/gut microbiota in conditions of metabolic disease [171,172,173,174,175,176]. This is in line with the emerging literature highlighting the potential benefits of pepper in improving chronic conditions, including those involving osteoarthritis, oropharyngeal dysphagia, digestion, hemodialysis, and neuromuscular fatigue (Table 1 and Table 2). However, the current literature supports the common use of pepper (including its active ingredients) in combination with other bioactive compounds, which could explain the synergistic effects in protecting against obesity-associated complications. Anyhow, current literature provides an important background for the clinical trials required to better investigate the therapeutic effects of black and red pepper, especially those involving individuals with metabolic syndrome.

## Figures and Tables

**Table 1:** An overview of human studies on the effects of black pepper (*Piper nigrum*) and its active ingredient, piperine, against diverse metabolic complications.

Author, Year	Country	Study Population	Intervention	Comparator (If Any)	Main Findings
Gregerse et al., 2013 [137]	Denmark	Individuals subjected to diet-induced thermogenesis (n = 22), with an average age of 25 years	Brunch meal with black pepper at 1.3 g, ginger (20 g), horseradish (8.3 g), and mustard (21 g) for 4 h	Placebo	Did not affect diet-induced thermogenesis; measurements of appetite and energy balance were also not affected
O’Connor et al., 2013 [138]	United States	Overweight women (n = 17), with an average age between 52–69 years	Black pepper at 1.5 g for 24 h	Placebo	Did not affect energy expenditure or respiratory quotient, including levels of glucose, insulin, catecholamines, and gut peptides
Rondanelli et al., 2013 [91]	Italy	Overweight individuals (n = 41), with an average age between 25 and 45 years	Two capsules per day, mainly containing *Camellia sinensis* decaffeinated dried extract (150 mg/cpr), microencapsulated oleoresin of *Capsicum annum* (7.5 mg/cpr), and piper nigrum dry extract, (3 mg/cpr) for 8 weeks	Placebo	Reduced obesity-related inflammatory metabolic dysfunction by ameliorating insulin resistance, improving the leptin/adiponectin ratio, respiratory quotient, and low-density lipoprotein (LDL) cholesterol levels
Hobbs et al., 2014 [139]	United States	Individuals with hypercholesterolemia (n = 19), with an average age between 18 and 80 years	Softgel that contained different active ingredients (such as bioflavonoids, vitamins, omega-3 fatty acids, and black pepper) for 30 days	Placebo	Reduced total cholesterol, low-density lipopolysaccharide, and triglyceride levels
Rofes et al., 2014 [140]	Spain	Individuals with oropharyngeal dysphagia (n = 40), with an average age between 74 and 78 years	Piperine at 1 mM or 150 μM during oropharyngeal swallow response	None	Alleviated oropharyngeal dysphagia by improving swallowing, with the time of laryngeal vestibule closure shortened at both concentrations
McCrea et al., 2015 [141]	United States	Overweight individuals given a high-fat meal (1000 kcal, 45 g fat) (n = 20), with an average age between 30 and 36 years	Capsule with a combination of spices (black pepper, cinnamon, cloves, garlic, ginger, oregano, paprika, rosemary, and turmeric) at 14.5 g for up to 210 min	Placebo	Reduced triglyceride levels, but did not have effects on glucose or insulin levels
Panahi et al., 2015 [142]	Iran	Individuals with metabolic syndrome (n = 50), with an average age between 36 and 53 years	Curcuminoids at 1 g, co-administered with piperine at 10 mg daily for 8 weeks	Placebo	Improved oxidative and inflammatory status by enhancing serum levels of superoxide dismutase (SOD) while reducing that of malonaldehyde (MDA), together with C-reactive protein
Gilardini et al., 2016 [143]	Italy	Obese females (n = 20), with an average age between 40 and 60 years	Formulation containing *Camellia sinensis*, titrated as > 60% polyphenols and > 40% in epigallocatechin-O-gallate, complexed with soy distearoylphosphatidylcholine and pure piperine (15 mg/dose) for 3 months	Placebo	Reduced body weight and fat mass
Zanzer et al., 2018 [144]	Sweden	Individuals receiving a meal rich in carbohydrates (n = 16), with an average age between 25 and 27 years	Black pepper-based beverage at 220 mL (20 mg gallic acid equivalent) up to 180 min	Placebo	Did not affect metabolic status. Also, the was no observed effects in the gastrointestinal well-being. However, there was suppression of hunger and improved satiety.
Mahmoudpour et al., 2019 [145]	Iran	Individuals with functional bloating (n = 36), with an average age between 20 and 50 years	Formulation containing *Trachyspermum ammi* (L.) Sprague seed, Zingiber officinale Roscoe. Rhizome, and *Piper nigrum* L. berry at 500 mg three times a day for 2 weeks	Placebo	Improved bloating status, including eructation, defecation, and borborygmus, better than dimethicone
Heidari-Beni et al., 2020 [146]	Iran	Individuals with chronic knee osteoarthritis (n = 30), with an average age between 35 and 75 years	Herbal formulation containing curcumin (300 mg), gingerols (7.5 mg), and piperine (3.75 mg), taken twice a day for 4 weeks	Naproxen at 250 mg	Potentially protected against chronic knee osteoporosis by reducing levels of prostaglandin E2
Oh et al., 2020 [147]	United States	Overweight or obese subjects (n = 12) given a high-fat meal (1000 kcal) (n = 20), with an average age between 40 and 65 years	Combination of spices (basil, bay leaf, black pepper, cinnamon, coriander, cumin, ginger, oregano, parsley, red pepper, rosemary, thyme, and turmeric) at 2 g for up to 4 h	Placebo	Alleviated high-fat-meal-induced postprandial interleukin (IL)-1β secretion
Pastor et al., 2020 [148]	Argentina	Individuals with metabolic syndrome (n = 22), with an average age between 63 and 73 years	Formulation containing resveratrol at 50 mg, piperine at 5 mg, and alpha tocopherol a 25 mg, with habitual treatment for 3 months	Placebo	Ameliorated inflammation by reducing levels of ferritin, ultrasensitive C-reactive protein, and oxygen consumption
Lindheimer et al., 2023 [149]	United States	Young adults with low energy (n = 40), with an average age between 18 and 34 years	Black pepper capsules twice a day at 0.504 g for 2 days	Rosemary at 0.425 g	Did not affect energy levels or fatigue feelings; however, rosemary induced a reduction in false alarm errors and mental fatigue at different time periods

**Table 2:** Clinical evidence of the effects of red pepper (*Capsicum annum*) and its active ingredient, capsaicin, against diverse metabolic complications.

Author, Year	Country	Study Population	Intervention	Comparator	Main Findings
Yoshioka et al., 1999 [150]	Canada	Healthy individuals given high-fat and high-carbohydrate meals (n = 23), with an average age between 23 and 41 years	Breakfast with red pepper at 10 g	None	Reduced appetite and subsequent protein and fat intake while also limiting energy intake
Lutgendorf et al., 2000 [151]	Denmark	Healthy individuals subjected to stressful conditions, with an average age between 21 and 33 years	Capsaicin at 510 mg for 10 days	Placebo	Ameliorated stressful related inflammation by enhancing relaxation; this was related to amendments in norepinephrine, heart rate, and systolic blood pressure during the experimental task
Belza and Jessen, 2005 [152]	Denmark	Overweight and obese individuals (n = 19), with an average age between 28 and 54 years	A tablet containing green tea extract at 250 mg, tyrosine at 203 mg, anhydrous caffeine at 25.4 mg, and capsaicin at 0.2 mg for 7 days	Placebo	Promoted a thermogenic effect through enhanced energy expenditure without raising the heart rate
Ahuja et al., 2006 [153]	Australia	Overweight individuals (n = 36), with an average age between 22 and 70 years	Chili blend (30 g/d; 55% cayenne chili) diet supplement for 4 weeks	None	Attenuated postprandial hyperinsulinemia
Inoue et al., 2007 [154]	Japan	Overweight individuals (n = 29), with an average age between 30 and 65 years	Capsinoids at 3 or 10 mg/kg for 4 weeks	Placebo	Promoted fat oxidation, and this positively correlated with the body mass index; further analysis showed that treatment enhanced energy expenditure and oxygen consumption
Snitker et al., 2008 [70]	United States	Overweight subjects (n = 41), with an average age between 30 and 60 years	Capsinoids at 6 mg for 12 weeks	Placebo	Safe and promoted fat oxidation
Chaiyasit et al., 2009 [155]	Thailand	Individuals subjected to oral glucose tolerance tests (n = 12), with an average age of 20–23 years	Capsaicin at 5 g for up to 120 min	None	Reduced plasma glucose levels and maintained insulin levels
Josse et al., 2010 [156]	Canada	Healthy subjects cycling at 55% VO_2_ peak, and for 30 min into recovery (n = 12), with an age between 21 and 27 years	Capsules of purified capsinoids at 10 mg, 30 min prior to exercise	None	Enhanced adrenergic activity, and energy expenditure, leading to a shift in substrate utilization toward lipid at rest but had little effect during exercise or recovery
Nieman et al., 2012 [157]	United States	Overweight and obese females (n = 31), with an average age between 40 and 75 years	A combination of red pepper spice at 1 g daily for 4 weeks	Received turmeric at 2.8 g	Did not affect inflammation and oxidative stress
Janssens et al., 2013 [158]	Netherlands	Healthy individuals subjected to 25% negative energy balance (n = 15), with an average age between 18 and 50 years	Capsaicin at 2.56 mg (1.03 g of red chili pepper, 39,050 SHU) with every meal for 36 h	Placebo	Supported negative energy balance by counteracting the unfavorable negative energy balance concomitant with a reduction in energy expenditure
Janssens et al., 2014 [159]	Netherlands	Healthy individuals (n = 15), with an average age between 18 and 50 years	Red chili pepper (containing capsaicin 2484 µ/g, nordihydrocapsaicin 278 µ/g, and dihydrocapsaicin 1440 µ/g) at 2.56 mg with every meal, mounting to daily total dose of 7.68 mg	None	Increased satiety and fullness, and partially prevented overeating when food intake was ad libitum; after dinner, treatment prevented the negative energy balance and desire to eat
Galgani et al., 2015 [160]	United States	Healthy subjects (n = 13), with an average age between 27 and 30 years	Gel capsules (containing capsinoids at 1, 3, 6 and 12 mg) up to 72 h	Placebo	Did not affect metabolic rate, non-protein respiratory quotient, blood pressure, or axillary temperature
Yuan et al., 2016 [161]	China	Women with gestational diabetes (n = 20), with an average age between 27 and 34 years	Capsaicin at 5 mg daily for 4 weeks	Placebo	Improved postprandial hyperglycemia and hyperinsulinemia, as well as fasting lipid metabolic disorders; in addition, the fasting serum levels of apolipoprotein B and calcitonin gene-related peptide increased compared to changes in glucose and insulin in the plasma
Joseph et al., 2021 [162]	India	Overweight subjects (n = 12), with an average age between 35 and 41 years	Capsifen (with 4 mg capsaicinoids/day) at 200 mg for 28 days	Placebo	Reduced body weight, body mass index, and appetite; results also affirmed the safety and tolerability of capsifen at the investigational dosage
Giuriato et al., 2022 [163]	Italy	Healthy males subjected to constant-load cycling exercise time-to-exhaustion trials (n = 10), with an average age between 19 and 26 years	Two capsules of capsaicin at 390 mg, during 72 h between sessions	Placebo	Alleviated neuromuscular fatigue through alterations in afferent signaling or neuromuscular relaxation kinetics
Silva-Santana et al., 2022 [164]	Brazil	Patients undergoing hemodialysis (n = 24), with an average age between 20 and 75 years	A combination of turmeric at 3 g and piperine at 2 mg daily for 12 weeks	Turmeric at 3 g/day	Combination treatment was superior in effectively modulating the status of oxidation and inflammation by reducing malonaldehyde and ferritin levels

## Data Availability

Data related to search strategy, study selection, and extraction items will be made available upon request after the manuscript is published.
